# Integrating Metagenomic and Culture-Based Techniques to Detect Foodborne Pathogens and Antimicrobial Resistance Genes in Malaysian Produce

**DOI:** 10.3390/foods14030352

**Published:** 2025-01-22

**Authors:** Jerrald Jia Weai Quek, Jun Leong Wong, Joon Liang Tan, Chew Chieng Yeo, Seow Hoon Saw

**Affiliations:** 1 Department of Allied Health Sciences, Faculty of Science, Universiti Tunku Abdul Rahman, Jalan Universiti, Bandar Barat, Kampar 31900, Perak, Malaysia; jerryquek@1utar.my; 2 Dr. Wu Lien-Teh Centre of Research in Communicable Diseases, M. Kandiah Faculty of Medicine and Health Sciences, Universiti Tunku Abdul Rahman, Jalan Sungai Long, Bandar Sungai Long Cheras, Kajang 43000, Selangor, Malaysia; drwongjl@gmail.com; 3 Department of Pre-Clinical Sciences, M. Kandiah Faculty of Medicine and Health Sciences, Universiti Tunku Abdul Rahman, Jalan Sungai Long, Bandar Sungai Long Cheras, Kajang 43000, Selangor, Malaysia; 4 Faculty of Information Science and Technology, Multimedia University, Jalan Ayer Keroh Lama, Bukit Beruang 75450, Melaka, Malaysia; jltan@mmu.edu.my; 5 Centre for Research in Infectious Diseases & Biotechnology (CeRIDB), Faculty of Medicine, Universiti Sultan Zainal Abidin, Jalan Sultan Mahmud, Kuala Terengganu 20400, Terengganu, Malaysia; chewchieng@gmail.com

**Keywords:** foodborne pathogens, metagenomics, culture-complementary PCR, pathogen detection, food safety, bacterial diversity, antimicrobial resistance genes (ARGs)

## Abstract

Foodborne illnesses pose a significant global health threat, often caused by pathogens like *Escherichia coli*, *Listeria monocytogenes*, and *Salmonella* spp. The emergence of antibiotic-resistant strains further exacerbates food safety challenges. This study combines shotgun metagenomics and culture-based approaches to detect foodborne pathogens and antimicrobial resistance genes (ARGs) in Malaysian produce and meats from the Kinta Valley region. A total of 27 samples comprising vegetables, meats, and fruits were analyzed. Metagenomics provided comprehensive microbial profiles, revealing diverse bacterial communities with species-level taxonomic resolution. Culture-based methods complemented these findings by identifying viable pathogens. Key foodborne pathogens were detected, with *Listeria monocytogenes* identified in meats and vegetables and *Shigella flexneri* detected inconsistently between the methods. ARGs analysis highlighted significant resistance to cephalosporins and penams, particularly in raw chicken and vegetable samples, underscoring the potential public health risks. While deli meats and fruits exhibited a lower antimicrobial resistance prevalence, resistant genes linked to *E. coli* and *Salmonella* strains were identified. Discrepancies between the methods suggest the need for integrated approaches to improve the pathogen detection accuracy. This study demonstrates the potential of metagenomics in advancing food safety research and supports its adoption as a complementary tool alongside culture-based methods for comprehensive foodborne pathogen surveillance and ARG profiling in Malaysian food systems.

## 1. Introduction

Foodborne illnesses are a significant global health concern, affecting an estimated 600 million people annually [1]. These diseases are primarily caused by pathogens such as *Escherichia coli*, *Campylobacter jejuni*, *Listeria monocytogenes*, *Salmonella* species (spp.), and *Shigella* spp., which enter the human body through the gastrointestinal tract via the consumption of contaminated or undercooked food and water [2,3]. Detecting these pathogens in food before consumption is critical for preventing outbreaks and safeguarding public health.

Traditional culture-based methods, considered the gold standard for pathogen detection, are valued for their sensitivity, cost-effectiveness, and capacity to yield both qualitative and quantitative data. However, these methods are labor-intensive, time-consuming, and require extensive molecular and biochemical validation [4,5]. Furthermore, challenges such as a limited detection speed, difficulty in resolving complex food matrices, and their inadequacy in real-time applications underscore the need for alternative approaches.

To address these limitations, various advanced technologies have emerged, including biosensors [6], molecular-based techniques such as the polymerase chain reaction (PCR) and next-generation sequencing (NGS) [7], Raman spectroscopy [8], and CRISPR-based diagnostics [9]. While these methods offer improved sensitivity, specificity, and rapid detection, practical challenges remain. For instance, their deployment in resource-limited settings, scalability for routine testing, and cost-effectiveness in real-world applications remain significant barriers. In this context, metagenomics, particularly shotgun sequencing, has gained traction as a robust tool capable of addressing some of these constraints by bypassing culture requirements and providing a comprehensive view of microbial communities [10]. Studies have demonstrated the utility of metagenomics in identifying pathogens during foodborne outbreaks and characterizing complex microbiomes in implicated samples [11,12].

In Malaysia, food safety research has revealed the presence of key foodborne pathogens such as *L. monocytogenes*, Shiga toxin-producing *E. coli* (STEC), *Salmonella* spp., and *C. jejuni* in various food products, raising concerns about the prevalence of antibiotic-resistant strains [13,14,15,16,17]. The overuse of antibiotics in agriculture and healthcare has exacerbated the emergence of antibiotic-resistant pathogens, complicating treatment efficacy and posing a critical public health challenge [17,18,19,20].

Despite these advances, the application of metagenomics in Malaysian food safety remains limited. This study investigates the feasibility of shotgun metagenomics for detecting foodborne pathogens in vegetables, meats, and fruits sourced from the Kinta Valley region, a major agricultural hub in Malaysia encompassing areas like Kampar, Gopeng, and Ipoh, known for its diverse produce and centralized markets [21,22,23,24]. Additionally, the study integrates culture-based PCR validation to enhance the reliability of metagenomic findings. A particular emphasis is placed on characterizing antimicrobial resistance genes (ARGs) within the detected pathogens to assess their potential public health impact. By addressing the limitations of existing detection technologies, this research aims to position shotgun metagenomics as a transformative tool for foodborne pathogen detection and to advance food safety management practices in Malaysia.

## 2. Materials and Methods

### 2.1. Sample Collection and Processing

This study analyzed nine sample types, categorized into three groups: vegetables (lettuce [L], spinach [S], cabbage [C]), meats (roasted chicken [RC], raw chicken [RC], deli meat [HM]), and fruits (honeydew [H], papaya [P], watermelon [W]). Each sample type was sourced from three vendors located in Kampar (K), Gopeng (G), and Ipoh (I) in the Kinta Valley district of Perak, Malaysia. Pooling samples offered a cost-effective approach that enhanced the likelihood of detecting low-abundance pathogens by consolidating resources and generalizing findings across a sample group [25]. Samples were chopped into smaller pieces, and 10 g from each vendor was pooled to form a 30 g composite sample for each type. These composite samples were then placed in stomacher bags consisting of 270 mL of a 0.85% saline solution. The bags were homogenized using a BagMixer (Interscience, Saint-Nom-la-Bretèche, France) for 2 min before being processed for genomic deoxyribonucleic acid (gDNA) extraction and culture-based analyses.

### 2.2. Culture of Foodborne Pathogens

The culture plate method was employed to validate the results obtained from metagenomic sequencing. The same samples analyzed via metagenomics were processed to isolate five different bacterial species, including *Campylobacter* spp., *Escherichia coli*, *Listeria* spp., *Salmonella* spp., and *Shigella* spp. Isolation procedures were conducted according to the Bacteriological Analytical Manual (BAM) recommendations (Table 1). Although *E. coli* is not considered as a pathogen, it remains as an important indicator for food safety. Each 30 g sample was added to a stomacher bag with 270 mL of specific enrichment media and homogenized. The mixtures were incubated under specific conditions. Following incubation, 500 μL of the enrichment broths was aliquoted into 1.5 mL microcentrifuge tubes and stored at −20 °C for subsequent boiled cell DNA extraction. Simultaneously, the enrichment broths were serially diluted ten-fold using a 0.85% saline solution. These serial dilutions were plated on selective agar plates using the spread plate method. The plates were incubated under specific conditions, and the colony morphology was observed (Table 1). Presumptive positive colonies were confirmed using boiled cell DNA extraction and conventional PCR.

### 2.3. Bacterial Spiking for Mock Community Creation

Bacteria spiking was performed using lettuce [L], raw chicken meat [RC], and honeydew [H] samples. The bacterial suspension’s Optical Density (OD) at 600 nm was measured using a UV–visible spectrophotometer (GENESYS 10S; Thermo Scientific, Waltham, MA, USA) and adjusted to 0.4, equivalent to approximately 2.04 × 10⁸ CFU/mL [26]. A 1 mL aliquot of each bacterial strain (*E. coli* ATCC BAA-197, *C. jejuni* ATCC 33560, *L. monocytogenes* ATCC 19115, *S. enterica* subspecies *enterica* serovar Typhimurium ATCC 700408, and *Sh. flexneri* ATCC 29903) was mixed with homogenized samples for 1 min before gDNA extraction. The step was repeated for validation with culture approaches. Additionally, 100 μL of the 10X diluted suspensions were spread on tryptic soy agar (TSA) plates and incubated as per the conditions in Table 1, and bacterial colonies were enumerated post-incubation.

### 2.4. Boiled Cell DNA Extraction and PCR

To extract DNA, 500 μL of the incubated enrichment broths (from Section 2.2) was centrifuged, and the resulting pellet was resuspended in 200 μL of distilled water. Concurrently, pure cultures of suspected bacterial colonies from tryptic soy agar (TSA) were picked using an inoculating loop and dissolved in 200 μL of distilled water in a new microcentrifuge tube. The suspensions were thoroughly mixed through vortexing. The cell suspensions were heated at 100 °C for 10 min in a heat block (Major Science Co. Ltd., Taoyuan, Taiwan). The tubes were then frozen at −20 °C for 10 min. After freezing, the tubes were centrifuged for 3 min, and the supernatant was carefully transferred into a new 1.5 mL microcentrifuge tube, with the pellet being discarded. This supernatant served as the DNA template for the PCR assay. The positive strains used in the PCR were *E. coli* ATCC BAA-197, *C. jejuni* ATCC 33560, *L. monocytogenes* ATCC 19115, *S. enterica* subspecies *enterica* serovar Typhimurium ATCC 700408, and *Sh. flexneri* ATCC 29903. Nine sets of forward and reverse primer pairs were used for the PCR detection of *Campylobacter* spp., *E. coli*, *Listeria* spp., *Salmonella* spp., and *Shigella* spp. (Table 2).

### 2.5. gDNA Extraction

The gDNA for metagenomic analysis was extracted using the Nucleospin Food Kit (Macherey-Nagel, Düren, Germany) with slight modification [11]. Briefly, the cell suspension from the samples (from Section 2.2) was centrifuged to collect the pellet, which was resuspended in 550 μL of preheated buffer CF (65 °C). After mixing for 15 s, 10 μL of proteinase K was added, and the suspension was incubated at 65 °C for 30 min. Following incubation, the mixture was centrifuged at 11,000× *g* for 10 min. The clear supernatant (400 μL) was transferred to a microcentrifuge tube. An equal volume of ethanol and buffer C4 (400 μL each) was added, vortexed for 30 s, and aliquoted onto a NucleoSpin^®^ Food Column. The tube was centrifuged at 11,000× *g* for 1 min to bind the DNA. The column was washed twice: first with 400 μL of buffer CQW, then with 700 μL of buffer C5, followed by an additional 200 μL of buffer C5 to ensure complete removal. Each wash was followed by centrifugation at 11,000× *g*, and the flow-through was discarded. For DNA elution, 50 μL of preheated elution buffer CE (70 °C) was pipetted onto the membrane and incubated at room temperature for 5 min before centrifuging it at 11,000× *g* for 1 min. This step was repeated to elute a total of 100 μL of DNA. The integrity of the extracted DNA was confirmed through agarose gel electrophoresis, showing a single band. The DNA concentration and purity were measured using a NanoDrop™ 1000 spectrophotometer (Thermo Scientific, Waltham, MA, USA), ensuring a concentration of at least 50 μg/mL, an A260/280 ratio between 1.8 and 2.0, and an A260/230 ratio of at least 1.8 before proceeding with library preparation.

### 2.6. DNA Library Preparation and Sequencing

The DNA samples were processed using the Illumina DNA Prep kit (Illumina, San Diego, CA, USA) according to the manufacturer’s recommendations. Briefly, 30 μL of the gDNA sample (from Section 2.4) was pipetted into a 96-well PCR plate and subjected to tagmentation at 55 °C for 15 min. Subsequent steps included stopping the tagmentation, purifying the tagmented DNA, amplifying the library, purifying the amplified library, and normalizing, pooling, and quantifying the library. Finally, sequencing was performed on the Novaseq 6000 platform with 150 bp paired end reads (Illumina, San Diego, CA, USA).

### 2.7. Bioinformatics Analysis

The raw sequencing data were uploaded to the Chan Zuckerberg (CZ) cloud-based metagenomic platform, utilizing the CZID Illumina mNGS Pipeline v8.3 (https://chanzuckerberg.zendesk.com/hc/en-us (accessed on 21 March 2024)). The quality control of the metagenomic reads was performed using the Fastp tool [30]. Rarefaction analysis was performed on all samples, demonstrating that the sequencing depth for each sample in this study was adequately represented and tabulated in Table 3. Rarefaction, a method first proposed by Sanders and refined by Hurlbert in the 1970s [31,32], adjusts for differences in library sizes across samples by subsampling reads to an even depth without replacement, enabling fair comparisons of alpha diversity metrics across ecosystems [33]. Host and duplicate reads were filtered using Bowtie2 and Hisat2, respectively [34,35]. The reads were then aligned to the National Centre for Biotechnology Information non-redundant (NCBI NR) database using Minimap2 and Diamond [36,37]. Short sequencing reads were assembled into contigs using SPAdes, with Bowtie2 associating reads with each assembled contig [38]. The assembled contigs were validated using the BLAST against the NCBI reference database. Data visualization, including heatmap generation, was conducted using the Python libraries Matplotlib (https://matplotlib.org/stable/users/index.html (accessed on 20 May 2024)), Seaborn (https://seaborn.pydata.org/ (accessed on 20 May 2024)) for plotting, and Pandas (https://pandas.pydata.org/docs/user_guide/index.html (accessed on 15 June 2024)) for data manipulation. To evaluate the agreement and discrepancies between metagenomics and culture-based methods for pathogen detection, two statistical analyses were performed: McNemar’s test and Cohen’s Kappa. These tests were carried out using IBM SPSS version 27.0.1, with the results data from Table 4 and Table 5 serving as the input.

An ARG pipeline was implemented following the completion of the data pre-processing workflow. Unlike the taxonomy assignment process, the assembly workflow for the ARG pipeline followed a distinct approach. Reads were mapped against the Comprehensive Antibiotic Resistance Database (CARD) using KMA (https://card.mcmaster.ca/, accessed on 20 May 2024), while contigs were assembled using SPAdes [38]. The assembled contigs were then analyzed for homology against the CARD using the Basic Local Alignment Search Tool (BLAST) (https://blast.ncbi.nlm.nih.gov/Blast.cgi, accessed on 20 May 2024). The results obtained were consolidated to provide ARG information, quality control (QC) matrices, and predictions for the pathogen of origin.

## 3. Results

### 3.1. Quality Control of Raw Reads

Rarefaction analysis confirmed that the sequencing depth was sufficient for each sample, with Q30 quality scores exceeding 90%, ensuring reliable downstream analysis [39]. The rarefaction curves reached a plateau state after a specific number of reads, leveling off at total read counts ranging approximately from 50 million to 300 million (Figure S1; Supplementary Materials). Read counts varied across the sample types, with vegetables yielding the highest count (352,638,836 reads), followed by fruits (306,922,112 reads) and meats (225,248,544 reads) (Table 3). Achieving high read counts, ideally at least 1 million reads per sample, is critical for accurate taxonomic and functional profiling [40].

### 3.2. Mock Community

#### 3.2.1. Comparison Between Metagenomic and Culture-Based Results

A mock community was prepared using five bacterial strains: *Campylobacter jejuni* ATCC 33560, *Escherichia coli* ATCC BAA-17, *Listeria monocytogenes* ATCC 19115, *Salmonella enterica* subspecies *enterica* serovar Typhimurium ATCC 700408, and *Shigella flexneri* ATCC 29903. These strains were introduced into vegetable, meat, and fruit samples.

The detection of these five pathogens using metagenomics analysis, alongside a bacterial culture complemented with PCR, are summarized in Table 4 and Table 5. *L. monocytogenes* was not successfully detected in vegetable and meat samples through metagenomics analysis but was identified using the culture-dependent method. The other four foodborne pathogens were detected in all three sample types with both detection techniques.

The performance of metagenomics compared to the culture method was evaluated using results data from Table 4 and Table 5. Metagenomics demonstrated a sensitivity of 68%, reflecting its capability to detect the majority of pathogens identified using the culture method, though some pathogens were not captured. The specificity was 99%, indicating that metagenomics rarely produced false positives. A positive predictive value (PPV) of 97% suggests that positive detections by metagenomics are highly reliable. Similarly, the negative predictive value (NPV) of 84% highlights that most negative results were accurate, though a small proportion of false negatives were present. These findings underscore that metagenomics is a highly specific method for pathogen detection, with opportunities for further optimization to improve the sensitivity.

The significant result from McNemar’s test (*p* < 0.001) underscores the discrepancies between the two methods, emphasizing the need for enhanced metagenomic workflows to improve the pathogen detection accuracy. Additionally, Cohen’s Kappa statistic was used to assess the agreement between metagenomics and culture-based methods for pathogen detection. The analysis yielded a Kappa value of 0.712 (*p* < 0.001), indicating substantial agreement between the two methods. This result demonstrates that while the methods are not perfectly aligned, their detection outcomes exhibit a high level of consistency beyond what would be expected by chance.

#### 3.2.2. Antimicrobial-Resistant Gene (ARG) Profiles

The three samples spiked with mock community bacteria were screened for ARGs, with the results presented in Table 6 and Table 7. Among the five bacteria in the mock community, two were identified as being associated with antimicrobial resistance: *E. coli* ATCC BAA-197 and *S.* Typhimurium ATCC 700408. *E. coli* ATCC BAA-197 is an extended-spectrum beta-lactamases (ESBL)-producing bacterium, while *S.* Typhimurium ATCC 700408 exhibits resistance to ampicillin, chloramphenicol, streptomycin, sulfonamide, and tetracycline.

The ARG profile of *E. coli* ATCC BAA-197 included seven distinct ARGs: *TEM-12*, *sul1*, *AAC(3)-IIe*, *SCO-1*, *APH(3′)-Ia*, *aadA1*, and *catA*. In contrast, *S.* Typhimurium ATCC 700408 carried four ARGs: *sul1*, *aadA16*, *floR*, and *CARB-2*. Metagenomic analysis revealed that most of the ARGs present in the mock community were detected in the spiked food samples. Specifically, five ARGs (*sul1*, *AAC(3)-IIe*, *SCO-1*, *APH(3′)-Ia*, and *floR*) were consistently identified across all three spiked food samples. However, *TEM-12*, *catA*, and *CARB-2* were not detected. Furthermore, the aminoglycoside nucleotidyltransferase genes *aadA1* and *aadA16* were absent in the vegetable and fruit samples, respectively. These findings warrant further validation through phenotypic assays for ARGs in future studies.

### 3.3. Metagenomic Analysis of Microbial Community

#### 3.3.1. Microbial Composition in Vegetables

**Figure 1:**
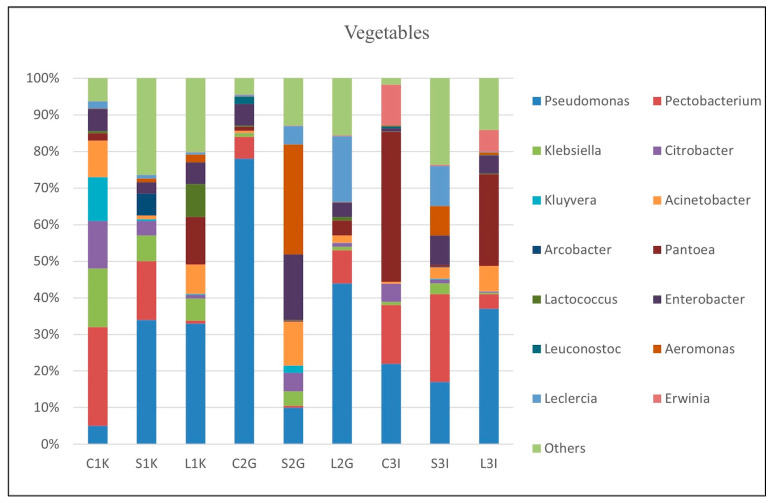
Major bacterial genera found in vegetable samples (*Pseudomonas*, *Pectobacterium*, *Enterobacter*, *Klebsiella*, *Pantoea*). C1K: Cabbage from Kampar; S1K: Chinese Spinach from Kampar; L1K: Lettuce from Kampar; C2G: Cabbage from Gopeng; S2G: Chinese Spinach from Gopeng; L2G: Lettuce from Gopeng; C3I: Cabbage from Ipoh; S3I: Chinese Spinach from Ipoh; L3I: Lettuce from Ipoh.

The bacterial composition at the genus level in vegetable samples is illustrated in Figure 1. *Pectobacterium* was consistently detected in all vegetable samples, albeit at varying abundances. In samples L1K and S2G, its relative abundance was below 1%, whereas in sample C1K, it accounted for 27%. Specific species such as *Pectobacterium brasiliense*, *P. polaris*, and *P. carotovorum* were particularly prevalent in six vegetable samples, including C1K, S1K, L2G, C3I, L3I, and S3I. Notably, two *Pectobacterium* species were among the dominant bacteria in sample L2G. *Pseudomonas* was another prevalent genus, detected across all samples and dominating the cabbage sample from Gopeng (C2G) with a relative abundance of 78%. Various *Pseudomonas* species were identified, including *P. otitidis*, *P. mendocina*, *P. azotoformans*, *P. oryzihabitans*, *P. putida*, *P. lurida*, *P. fluorescens*, and *P. simiae* (Table S1; Supplementary Materials). Sample C2G exhibited a predominance of four distinct *Pseudomonas* species, while *P. otitidis*, *P. mendocina*, and *P. azotoformans* were particularly abundant in samples L1K, S1K, and C2G, respectively. *Klebsiella* was detected in all samples, with relative abundances ranging from less than 1% to 16%. Pathogenic *Klebsiella* species such as *K. pneumoniae* were detected but at lower levels. Similarly, *Enterobacter*, including pathogenic species such as *Enterobacter cloacae* and *E. roggenkampii*, was prominent in samples S2G, L3I, and S3I, with *E. cloacae* being especially abundant in sample S2G.

Other genera such as *Citrobacter*, *Kluyvera*, *Acinetobacter*, *Pantoea*, and *Leclercia* were also detected in varying proportions, with each genus contributing significantly to the microbial diversity. The category “Others” accounted for 4% to 26% of the relative abundance, highlighting a diverse range of less common bacterial genera. This comprehensive profiling of bacterial communities underscores the diversity and complexity of microbial populations present in vegetable samples, with potential implications for food safety, spoilage, and agricultural practices.

#### 3.3.2. Microbial Composition in Meats

**Figure 2:**
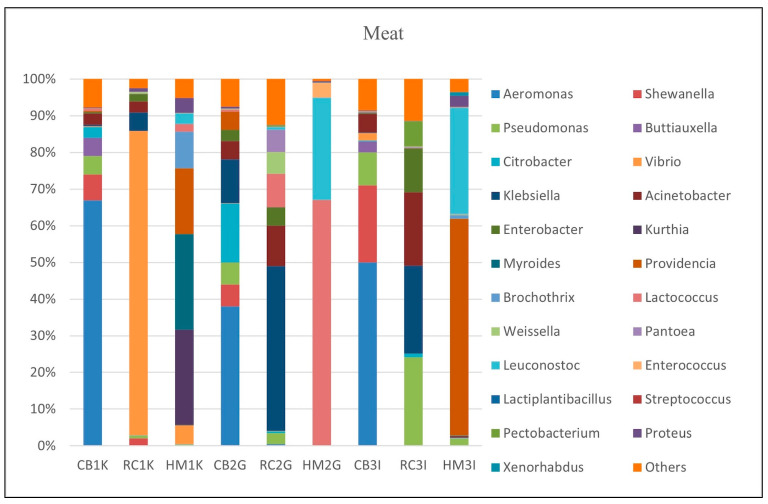
Major bacterial genera found in meat samples (*Aeromonas*, *Pseudomonas*, *Vibrio*, *Lactococcus*, *Leuconostoc*). CB1K: Raw chicken from Kampar; RC1K: Roasted chicken from Kampar; HM1K: Deli meat from Kampar; CB2G: Raw chicken from Gopeng; RC2G: Roasted chicken from Gopeng; HM2G: Deli meat from Gopeng; CB3I: Raw chicken from Ipoh; RC3I: Roasted chicken from Ipoh; HM3I: Deli meat from Ipoh.

At the genus level, the bacterial diversity in meat samples revealed notable variations across the six analyzed samples (CB1K, RC1K, CB2G, RC2G, CB3I, RC3I), as illustrated in Figure 2. *Aeromonas* exhibited wide-ranging abundances, from 0.01% in RC1K to 67% in CB1K, and was particularly prominent in raw chicken samples. *Pseudomonas* was detected in all meat samples except for the deli meat sample HM2G. Other significant genera included *Vibrio* and *Lactococcus*, which were dominant in RC1K (83%) and HM2G (67%), respectively. The “Others” category contributed to between 0.5% and 12% of the microbial communities, with the lowest percentage in HM2G and the highest in RC2G.

The five most abundant bacterial species in the meat samples (Table S2; Supplementary Materials), including *Aeromonas* species, such as *A. veronii*, *A. salmonicida*, and *A. hydrophila*, were predominant in the raw chicken samples (CB1K, CB2G, CB3I). *A. veronii* was the most abundant species in CB1K and CB2G and the second most abundant in CB3I, while *A. hydrophila* was particularly prevalent in CB3I. In roasted chicken samples (RC1K, RC2G, RC3I), *Acinetobacter* species, such as *Ac. baumannii*, *Ac. calcoaceticus*, *Ac. johnsonii*, and *Ac. tandoii*, were frequently identified. Conversely, the deli meat samples (HM2G, HM3I) were dominated by *Leuconostoc* species, including *Le. citreum*, *Le. carnosum*, and *Le. mesenteroides*. *Le. citreum* and *Le. carnosum* were the most abundant in HM2G, while HM3I displayed a higher abundance of *Le. carnosum* and *Le. mesenteroides*.

#### 3.3.3. Microbial Composition in Fruits

**Figure 3:**
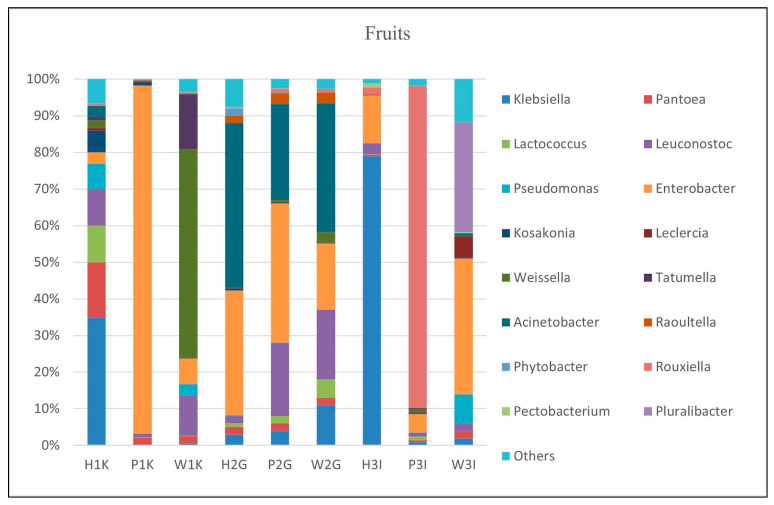
Major bacterial genera found in fruit samples (*Enterobacter*, *Leuconostoc*, *Rouxiella*, *Pantoea*, *Acinetobacter*). H1K: Honeydew from Kampar; P1K: Papaya from Kampar; W1K: Watermelon from Kampar; H2G: Honeydew from Gopeng; P2G: Papaya from Gopeng; W2G: Watermelon from Gopeng; H3I: Honeydew from Ipoh; P3I: Papaya from Ipoh; W3I: Watermelon from Ipoh.

The microbial composition of fruit samples revealed distinct bacterial profiles, with the five most abundant genera depicted in Figure 3. *Leuconostoc* was consistently present across all fruit samples, with relative abundances ranging from 1% to 20%. The genus *Enterobacter* showed a wide abundance range (3–95%), being the most dominant in the papaya sample from Kampar (P1K), in which it had a 95% relative abundance. *Rouxiella* was notably dominant in sample P3I, comprising 88% of the microbial community. *Pantoea* was detected in all fruit samples, with abundances ranging from 0.4% to 15%. The “Others” category contributed to between 0.2% and 12% of the microbial communities, a lower range than that observed in vegetable samples.

*Enterobacter* species (*En. cloacae*, *En. hormaechei*, *En. asburiae*, and *En. roggenkampii*) were abundant in most fruit samples, except for H1K (Table S3; Supplementary Materials). Sample H2G exhibited significant levels of *En. asburiae*, *En. roggenkampii*, and *En. cloacae*. Additionally, fruit samples from Gopeng (W2G, H2G, P2G) displayed consistently high abundances of *Acinetobacter* species, such as *Ac. seifertii* and *Ac. baumannii*. *Leuconostoc* species (*Le. lactis* and *Le. garlicum*) were abundant in six fruit samples (W1K, H1K, P1K, W2G, P2G, P3I). Notably, *Le. lactis* was prevalent in all samples except W1K, where *Le. garlicum* showed higher levels.

### 3.4. Comparative Analysis of Foodborne Pathogens in Vegetables, Meat, and Fruits Using Metagenomics and Culture Methods

**Figure 4:**
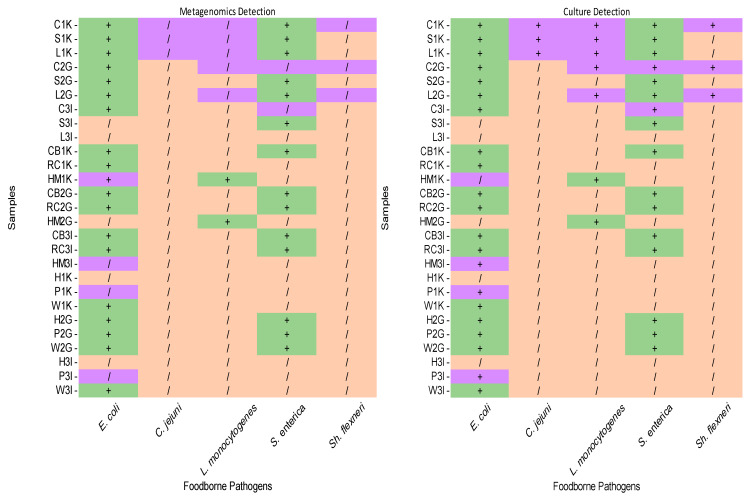
The detection of foodborne pathogens in vegetable, meat, and fruit samples using metagenomics and culture approaches (green represents detection by both approaches; orange indicates detection by neither approach; purple shows a discrepancy in the approaches). +: positive detection; /: no detection.

The detection of foodborne pathogens in vegetable, meat, and fruit samples using metagenomics and culture methods complemented by PCR is detailed in Figure 4. Although *E. coli* is not considered as a pathogen, some of its strains are potentially pathogenic, and the bacterium is an important indicator of hygiene levels in food safety [41]. Thus, this study included *E. coli* in the comparative analysis.

#### 3.4.1. Vegetables

Both techniques detected *E. coli* and *S.* Typhimurium in all vegetable samples acquired from the three different locations. *C. jejuni* was only detected in vegetables from Kampar using both techniques. Cabbage samples from Kampar and Gopeng (C1K, C2G) and a lettuce sample from Gopeng (L2G) tested positive for *Sh. flexneri* using both techniques, but it was absent in the remaining samples. *L. monocytogenes* was detected in four samples (C1K, S1K, L1K, C2G) using both approaches and in sample L2G using culture methods only.

#### 3.4.2. Meats

Neither approach detected *C. jejuni* in meat samples. Two deli meat samples (HM1K, HM2G) were positive for *L. monocytogenes*. *S.* Typhimurium was detected in all meat samples except the deli meat samples (HM1K, HM2G, HM3I). Culture methods detected *E. coli* in sample HM3I and *Sh. flexneri* in sample RC2G, which were not detected by metagenomics. Both techniques detected *E. coli* in six samples (CB1K, RC1K, CB2G, RC2G, CB3I, RC3I) and *Sh. flexneri* in two samples (CB2G, RC3I).

#### 3.4.3. Fruits

*E. coli* was detected in all fruit samples using both techniques, whereas *C. jejuni* was not detected in any fruit samples. *L. monocytogenes* was not detected in any fruit samples using either method. Metagenomics detected *S.* Typhimurium in all fruit samples, while culture methods found the watermelon sample from Kampar to be negative. The papaya sample from Ipoh was positive for *Sh. flexneri* according to culture methods but not according to metagenomics. Discrepancies were noted in *Sh. flexneri* detection, with one sample out of nine being inconsistent. Notably, sample W1K was positive for metagenomics but negative for culture analysis.

### 3.5. Antimicrobial Resistance Gene (ARG) Profiles

**Figure 5:**
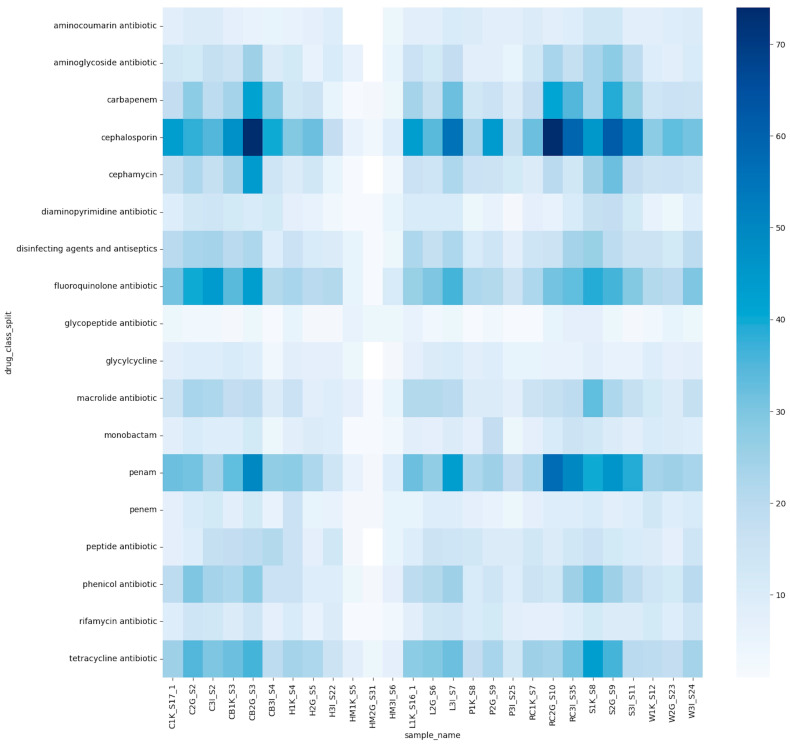
A heatmap on the distribution of antimicrobial resistance (AMR) genes among all 27,396 samples analyzed with metagenomics. The darker shade of blue indicates a higher abundance, whereas the lighter shade of blue represents a lower abundance.

Metagenomics analysis was employed to predict antimicrobial resistance profiles across 27 food samples, categorizing resistance genes by antibiotic classes (Figure 5). The darker color gradients in the figure represent a higher abundance of specific resistance genes. Resistance genes associated with aminocoumarin, glycylcycline, and phosphonic acid were the least prevalent among all samples.

Approximately half of the samples exhibited resistance genes linked to lincosamide, macrolide, peptide, phenicol, and rifamycin antibiotics. In contrast, resistance genes associated with aminoglycoside, carbapenem, cephamycin, diaminopyrimidine, disinfectants, fluoroquinolone, monobactam, penem, sulfonamide, and tetracycline were more commonly detected, highlighting a broader presence across the sample set.

The highest occurrences of resistance genes were observed for the cephalosporin and penam antibiotic classes. Notably, 83% of the cephalosporin resistance genes were predicted to be linked to *Klebsiella aerogenes* and *Klebsiella pneumoniae*. Penam resistance genes were predominantly associated with bacteria such as *Acinetobacter* spp. and *Escherichia coli*.

#### 3.5.1. High ARG Abundance

The raw chicken sample from Gopeng (CB2G) and spinach sample from Kampar (S1K) displayed a higher abundance of resistance genes compared to other samples. Specifically, S1K showed darker regions for fluoroquinolone, macrolide, and phenicol resistance, indicating elevated resistance levels to these antibiotic classes.

#### 3.5.2. Low ARG Abundance

Samples with a lower ARG abundance included deli meat and certain fruit samples, such as HM2G, HM3I, L2G, P1K, P3I, W1K, W2G, and W3I. Deli meat samples (HM1K and HM2G) exhibited the lowest occurrence of resistance genes across the dataset.

#### 3.5.3. Fluoroquinolone Resistance

Darker shaded regions for fluoroquinolone resistance were prominent in samples C3I, CB2G, S1K, and S2G, indicating a higher prevalence of resistance genes associated with this antibiotic class.

These results provide insights into the variability of ARG distribution across different food matrices and geographical sources, suggesting potential risks and the need for targeted interventions to mitigate the spread of antimicrobial resistance in food systems.

## 4. Discussion

### 4.1. Mock Community

#### 4.1.1. Metagenomic and Culture-Based Methods

Metagenomic analysis successfully detected *L. monocytogenes* in fruit samples but not in vegetables or meat, in contrast to culture-dependent methods (Table 4 and Table 5). This discrepancy may be attributed to the higher detection limits required for metagenomic analysis, where a low pathogen abundance can result in non-detection. Additionally, the target pathogen may have been excluded due to the filtering of low-quality reads [31,32]. Nonetheless, both methods consistently identified four other foodborne pathogens across vegetables, meats, and fruits (Table 4 and Table 5).

#### 4.1.2. ARG Profiles

The screening of ARGs focused on identifying resistance profiles within the mock community. Beta-lactamase-associated genes, such as *SCO-1*, were detected in all spiked food samples (Table 6). Additionally, ARGs linked to sulfonamide and aminoglycoside were identified. Two ARGs (*sul1* and *floR*) from *S.* Typhimurium ATCC 700408 were detected in all food samples (Table 7). Although the carbenicillin-hydrolyzing class A beta-lactamase gene (*CARB-2*) was not detected, related ARGs such as *CARB-1* and *CARB-3* were identified.

The overall accuracy of ARG detection in the mock community was 67%. According to a study by Mahfouz and colleagues [42], while the Comprehensive Antibiotic Resistance Database (CARD) exhibited a low very major error rate of 1.17%, its accuracy could be further enhanced by improving the completeness and quality of its curated data. Overall, the majority of ARGs present in the ATCC strains were successfully identified through metagenomics. These findings underscore the potential of metagenomic analysis for detecting ARGs. Although Malaysian researchers have investigated and validated the application of metagenomics for ARG detection, its adoption within the food safety industry remains limited [43,44].

### 4.2. Metagenomic Analysis of Microbial Community

#### 4.2.1. Microbial Composition in Vegetables

The microbial communities in vegetable samples were dominated by genera within Proteobacteria, with notable contributions from *Pectobacterium*, *Pseudomonas*, *Klebsiella*, and *Enterobacter* (Figure 1). *Pectobacterium* species (*P. brasiliense*, *P. polaris*, and *P. carotovorum*) are well-known plant pathogens, producing cell wall-degrading enzymes that lead to tissue maceration and rot in vegetables like cabbage, celery, and leeks [45,46,47,48]. The prevalence of *Pectobacterium* species in multiple samples, including L2G and C1K, highlights their significant role in vegetable spoilage and postharvest losses.

*Pseudomonas* spp., such as *P. otitidis*, *P. mendocina*, and *P. azotoformans* were prominent in different samples, suggesting niche adaptations [49,50]. While many *Pseudomonas* species contribute to plant health through nutrient production, others, including *P. otitidis* and *P. oryzihabitans*, exhibit pathogenic traits linked to agricultural diseases and human infections [51,52]. On the other hand, *Klebsiella pneumoniae* is a potential contaminant with implications for public health due to its role in nosocomial infections and increasing antibiotic resistance. Similarly, *Enterobacter cloacae* is an emerging concern for its resistance to third-generation antibiotics; it also demonstrates beneficial plant growth-promoting activities, including nitrogen fixation and phosphate solubilization, which are valuable for sustainable agriculture [53].

Other genera, including *Citrobacter*, *Kluyvera*, *Acinetobacter*, *Pantoea*, and *Leclercia*, contributed to the microbial diversity, albeit at lower abundances. These genera, along with the “Others” category, reflect the complexity of vegetable-associated microbial communities, encompassing both spoilage agents and potentially beneficial microorganisms (Figure 1).

The consistent detection of these genera across samples underscores the dual role of the vegetable microbiome in promoting plant health and presenting food safety challenges. Addressing contamination risks, such as those posed by irrigation water and postharvest handling, while leveraging beneficial microbial traits, could improve agricultural productivity and reduce spoilage and health risks [54].

#### 4.2.2. Microbial Composition in Meats

The bacterial composition of meat samples reflects distinct microbial profiles influenced by processing methods and meat types, with significant implications for food safety, spoilage, and public health (Figure 2).

*Aeromonas* species, notably *A. veronii*, *A. hydrophila*, and *A. salmonicida*, were highly abundant in raw chicken samples (Figure 2), indicating their critical role in meat spoilage and contamination. These bacteria, commonly found in freshwater environments, possess virulence factors such as endotoxins, cytotoxins, and proteolytic enzymes, contributing to their pathogenicity and spoilage potential [55]. For instance, *A. salmonicida* is closely associated with spoilage in chilled meats due to its proteolytic activity [56]. The presence of these species highlights the vulnerability of raw chicken to contamination, emphasizing the need for stringent hygiene practices during processing and storage.

*Pseudomonas*, another prominent genus, was detected in most samples, reflecting its ubiquity in meat environments. Known for its high spoilage potential, *P. aeruginosa* can survive in raw, processed, and vacuum-packaged meats, even post-thermal processing [57,58]. Its persistence underscores its adaptability and the challenges it poses for ensuring meat quality and safety. *Acinetobacter* species, prevalent in roasted chicken samples, present significant concerns due to their ability to harbor antibiotic resistance genes. Strains such as *Ac. johnsonii* and *Ac. baumannii* are known to produce biofilms and secrete proteases, contributing to spoilage and posing public health risks [59]. *A. baumannii* is a critical top-priority pathogen listed by the World Health Organization [60]. The identification of these strains in food highlights the potential of meat products as reservoirs for antibiotic-resistant bacteria [61].

In deli meats, *Leuconostoc* species (*Le. citreum*, *Le. carnosum*, and *Le. mesenteroides*) were dominant (Figure 2). These bacteria demonstrate a dual role in meat products. While their antimicrobial properties, attributed to organic acid production, offer potential as bio-preservatives, their spoilage traits, including exopolysaccharide production, can lead to undesirable changes such as slime formation and texture alteration [62]. The prevalence of *Le. carnosum* in refrigerated and high-salt environments explains its abundance in processed deli meats and underscores its adaptability to such conditions [63]. The “Others” category, representing diverse and less abundant bacterial genera, further underscores the complexity of microbial communities in meat samples. While some of these bacteria may contribute to spoilage, others could have beneficial roles, warranting further investigation into their functional characteristics.

This study highlights the critical need for targeted interventions to mitigate contamination risks in meat processing and storage. Improved hygiene practices, effective cold chain management, and the potential utilization of beneficial bacteria as natural preservatives could collectively enhance meat safety and quality while reducing spoilage and health risks.

#### 4.2.3. Microbial Composition in Fruits

The microbial diversity in fruit samples highlights the significant roles of Proteobacteria and Firmicutes in shaping the quality, safety, and potential risks associated with fresh produce (Figure 3).

Proteobacteria, particularly *Enterobacter* species, are integral to the fruit microbiome, influencing both beneficial and detrimental outcomes. *En. cloacae* are linked to melon necrosis, where its fermentative activity can cause rind damage and fruit bursting [64,65]. Conversely, other *Enterobacter* species, such as *En. hormaechei* and *En. asburiae*, can benefit fruit crops. *En. hormaechei* enhances plant growth by solubilizing essential nutrients like potassium and calcium [66], while *En. asburiae* has shown potential in inhibiting harmful pathogens in cantaloupes [67]. These findings underscore the dual roles of Enterobacter species in fruit ecosystems, balancing spoilage risks with potential agricultural benefits.

The consistent presence of *Acinetobacter* species, particularly *Ac. seifertii* and *Ac. baumannii*, in Gopeng fruit samples is noteworthy (Figure 3). While *Ac. baumannii* is known for its biofilm formation and environmental resilience, its association with agricultural products highlights its adaptability [68]. Similarly, *Ac. seifertii*, typically implicated in serious infections, is increasingly recognized in agricultural contexts, warranting further investigation into its transmission routes and implications for food safety [69].

Firmicutes, represented by *Leuconostoc* species, play diverse roles in fruit ecosystems. *Le. lactis* contributes to fruit preservation by reducing weight loss and decay, particularly in strawberries, through sugar breakdown and the production of fermentation by-products [63]. This characteristic also makes it integral to the fermentation of plant-based foods. In contrast, *Le. garlicum* thrives in extreme environments, such as garlic surfaces, due to its resilience and enzymatic capabilities [70]. While its role in fruits remains underexplored, its detection in papaya and watermelon samples underscores its adaptability to diverse plant matrices.

The diversity within the “Others” category suggests the presence of less dominant but potentially influential bacterial taxa. Although these taxa comprise a smaller proportion of the microbial community, their functional contributions, whether in spoilage, preservation, or plant–microbe interactions, merit further exploration.

This study emphasizes the intricate interplay between microbial diversity and fruit quality. Targeted microbial management strategies, including improved postharvest handling and the potential biocontrol applications of beneficial microbes like *Le. lactis* and *En. hormaechei*, could mitigate risks while enhancing the nutritional and sensory attributes of fruits.

### 4.3. Comparison Between Metagenomic and Culture-Based Results

The detection outcomes for *E. coli*, *C. jejuni*, *S. Tyhimurium*, and *Sh. flexneri* in vegetable samples showed consistency between metagenomics and culture methods (Figure 4). However, a notable discrepancy emerged in the detection of *L. monocytogenes,* where metagenomics failed to identify its presence in one lettuce sample. Previous studies using spiked spinach samples reported a detection accuracy of 94% for *E. coli* using metagenomic sequencing [71]. In contrast, our study demonstrated higher detection accuracy for *E. coli* in vegetables, but the accuracy for *L. monocytogenes* was limited to 89%. Discrepancies were more pronounced in meat samples, where the detection accuracy was 89% for both *E. coli* and *Sh. flexneri*. Similarly, in fruit samples, the detection accuracy was 89% for *S.* Typhimurium and *Sh. flexneri.* Factors such as the number of pathogens present, the complexity of the food matrix, and the interference from host DNA further exacerbate the challenges of pathogen identification through metagenomics, as previously noted in food production environments [72].

### 4.4. Foodborne Pathogen Detection Consistency

*C. jejuni* showed the highest detection consistency, being consistently detected in all 27 samples across both approaches (Figure 4). *E. coli*, *L. monocytogenes,* and *S.* Typhimurium had a detection accuracy of 96% (26 out of 27 samples), while *Sh. flexneri* had the lowest accuracy at 93% (25 out of 27 samples). The inconsistency in the results may stem from factors such as the sensitivity of metagenomics, the detection of non-viable bacteria, and the sample complexity. Previous studies have achieved detection sensitivity for *Shiga* toxin-producing *E. coli* (STEC) at 10 colony forming units (CFUs) per 100 g of a sample [73]. However, this study’s methodology, which omitted the enrichment step, might have affected the sensitivity [74].

As for the unexpected inverse relationship between *Salmonella*-positive and *Listeria*-positive samples, it may be attributed to the competition between microbial species in the food matrix or differences in environmental niches that favor the survival of one pathogen over the other. While the sampling was conducted randomly, these biological and environmental factors could have influenced the microbial composition of the samples.

Metagenomics does not discriminate between viable and non-viable bacteria, thus potentially detecting dead bacteria, which can be useful for source-tracking investigations [75]. While DNA-binding dyes like propidium monoazide can mitigate this issue, they were not used in this study due to their impact on sequencing results [76]. Additionally, complex background microbiota from human and environmental sources can interfere with the sensitivity and results [77].

### 4.5. Antimicrobial Resistance Gene (ARG) Profiles

The elevated resistance to cephalosporins and penams observed in foodborne pathogens is linked to several interconnected factors, including the widespread use of these antibiotics in human medicine and agriculture (Figure 5). This practice facilitates the emergence and proliferation of resistant strains, as seen with *E. coli* and *Salmonella*, which frequently carry resistance to β-lactam antibiotics such as cephalosporins [78]. Agricultural antibiotic usage exacerbates resistance development, further contributing to the transmission of ARGs through the food chain. Surveillance data highlight significant resistance trends, such as resistance to quinolones and cephalosporins in *Salmonella enterica* and to quinolones and macrolides in *Campylobacter* spp., underscoring the critical need to regulate antibiotic use in animal farming to mitigate resistance dissemination [79,80].

#### 4.5.1. Variability of ARGs by Sample Type

Deli meats and fruits generally exhibited lower occurrences of ARGs compared to vegetables and raw chicken. This may be attributed to the stringent hygiene and safety measures often applied during the processing and handling of deli meats. Deli meats have been processed, and the curing process (including the addition of preservatives and a high salt content) could lower the survival chances of these ARG pathogens [81,82,83,84].

Studies using high-throughput techniques and qPCR have shown that while tetracycline-resistant (TR) *Enterobacteriaceae* can be detected in meat samples, their abundance is often reduced in products subjected to robust safety protocols. Conversely, environmental sources like river sediments and wastewater outputs tend to harbor a higher diversity and prevalence of ARGs [40,85].

Raw chicken and vegetable samples, however, demonstrated substantial resistance, particularly to cephalosporins and penams. In Malaysia, a notable 25% of *S. enterica* isolates have shown resistance to ceftriaxone, a third-generation cephalosporin [86]. Similarly, cephalosporin-resistant *Enterobacterales*, including *E. coli* and *K. pneumoniae*, have been identified in raw meat globally. In the U.S., 5.6–10.8% of meat samples tested positive for resistant strains, further underscoring the role of meat as a significant reservoir of ARGs [87]. Additionally, *Listeria* isolates from Malaysian vegetable farms and retail markets exhibited total resistance to penicillin G, while *Salmonella* isolates from poultry and vegetable farms displayed widespread resistance to ampicillin and amoxicillin [88,89].

#### 4.5.2. Resistance Trends in Specific Antibiotic Classes

Resistance genes associated with aminocoumarin, glycylcycline, and phosphonic acid classes were among the least prevalent in this study. For aminocoumarins, their limited use and specificity against bacterial gyrase restrict their application, which may explain the low occurrence [90]. Glycylcycline resistance, including tigecycline resistance genes, was similarly rare. A study in China found a low prevalence (0.65%) of tigecycline resistance in *E. coli* from food sources, although higher levels (9.35%) were observed in *Enterobacterales* from porcine origins, highlighting the variability of resistance across bacterial sources [91,92].

Phosphonic acid resistance, particularly fosfomycin resistance, was also minimally detected. However, its growing prevalence in *Campylobacter* and *Salmonella* is concerning, as these pathogens can transmit resistance to humans through food, complicating the treatment of infections [93,94]. Despite its low occurrence in this study, fosfomycin remains a valuable antibiotic for treating certain infections, emphasizing the importance of continuous monitoring and judicious antibiotic use to prevent resistance escalation.

#### 4.5.3. Implications and Future Considerations

The observed resistance patterns reflect the complex interplay between antibiotic use, agricultural practices, and food safety regulations. The widespread resistance to cephalosporins and penams necessitates stricter controls on antibiotic usage, particularly in agricultural settings. Simultaneously, the lower prevalence of resistance in less utilized antibiotic classes such as glycylcyclines and phosphonic acids highlights potential areas for strategic intervention to safeguard these antibiotics. Overall, these findings underscore the importance of an integrated “One Health” approach to addressing antimicrobial resistance through enhanced surveillance, judicious antibiotic use, and improved hygiene practices across the food production chain.

### 4.6. Novelty and Impact on Malaysian Food Safety Research

The integration of metagenomics with culture-based methods, while explored globally, has not been extensively applied in the Malaysian context, where unique dietary habits, agricultural practices, and environmental conditions shape the microbial profile of food [95,96]. This study provides novel insights by focusing on foodborne pathogens and ARGs in the Kinta Valley, an agricultural hub with diverse produce and centralized markets. By characterizing microbial communities and ARGs in vegetables, meats, and fruits specific to this region, the study offers a localized perspective that is crucial for understanding and managing food safety challenges in Malaysia.

### 4.7. Limitations and Future Directions

Nonetheless, challenges remain, including the absence of standardized procedures and approaches, which often result in discrepancies among studies. Metagenomics predominantly provides data in terms of the relative abundance, limiting its ability to directly correlate with quantitative measures like colony forming units (CFUs) [7]. Additionally, current metagenomic techniques cannot differentiate viable and non-viable organisms without supplementary methods such as DNA-binding dyes [76]. Constrained sample sizes, often dictated by the high costs of metagenomic analyses, further necessitate the aggregation of samples and restrict the geographical scope of sampling. This study was based on 27 samples from three geographical locations, which may limit the generalizability of the findings. Expanding the study’s geographical and sample scopes would add depth to the overall findings in the near future.

### 4.8. Shotgun Metagenomics for Foodborne Pathogen Detection in Malaysia

Despite these limitations, shotgun metagenomics has proven effective in detecting foodborne pathogens in vegetables, meats, and fruits, providing species-level taxonomic resolution. This study demonstrates the capability of metagenomics to highlight significant food safety concerns in Malaysia and underscores its potential for widespread application in the nation’s food safety sector. By validating metagenomic findings with culture-based PCR, this research enhanced the robustness and reliability of pathogen detection, setting a precedent for future studies.

Future investigations could complement metagenomics with quantitative methods like qPCR, cultures, and flow cytometry to overcome existing limitations and improve the detection accuracy and reliability [97]. As technological advancements continue to reduce costs, the integration of metagenomics into standard workflows for food safety monitoring is becoming increasingly feasible. Despite current obstacles, metagenomics remains a powerful tool for simultaneous pathogen detection and microbial community profiling, making it indispensable for advancing food safety and security in Malaysia. This combination of a regional focus, methodological rigor, and forward-looking recommendations affirms this manuscript’s contribution and its potential to inform public health interventions.

## 5. Conclusions

This study highlights the potential of shotgun metagenomics as a powerful tool for food safety management. By achieving the key objectives, it demonstrated the feasibility of using metagenomics to detect foodborne pathogens with species-level accuracy, profile microbial communities in various food samples, and predict antimicrobial resistance genes (ARGs). While metagenomics showed an overall detection accuracy of 83% for pathogens, distinct microbial profiles and trends were observed across vegetables, meats, and fruits, with notable genera including *Klebsiella*, *Lactococcus*, and *Enterobacter*. Additionally, ARGs linked to cephalosporin and penam antibiotics were predominantly found in raw chicken and vegetable samples. This dual capability of shotgun metagenomics—to profile microbial communities and predict ARGs—offers valuable insights for advancing foodborne pathogen detection and enhancing food safety practices.

## Figures and Tables

**Table 1:** Method of enrichment, incubation, and isolation of five foodborne pathogens.

Bacterial spp.	Enrichment Phase		Plating Phase		Colony Morphology
	Media Broth	Incubation Conditions	Agar	Incubation Conditions	
*Campylobacter*	Bolton	Anaerobic chamber with microaerobic gas-generating pouch (microaerobic atmosphere (10% CO_2_, 5% O_2_, and 85% N_2_)), 42 °C, 48 h	BFC	Anaerobic chamber with anaerobic gas-generating pouch, 42 °C, 48 h	Gray and flat with or without green hue
*Escherichia coli*	EC	42 °C, 48 h	EMB	42 °C, 48 h	Green metallic sheen with dark center
*Listeria*	BLEB	30 °C, 48 h	PALCAM	30 °C, 48 h	Gray-green with black halo
*Salmonella*	BPW; after 24 h incubation, aliquoted 1 mL into 9 mL RV broth	BPW at 37 °C, 24 h RV at 37 °C, 24 h	XLD	37 °C, 24 h	Pink or red with black center
*Shigella*	*Shigella* broth	Anaerobic chamber with anaerobic gas-generating pouch, 42 °C, 20 h	MAC	35 °C, 20 h	Pale or colorless

Footnotes: BFC: Blood-free *Campylobacter* agar; EC: *Escherichia coli* broth; EMB: Eosin–methylene blue agar; BLEB: Buffered *Listeria* enrichment broth; BPW: Buffered peptone water; XLD: Xylose–lysine–deoxycholate agar; RV: Rappaport–Vassiliadis broth; MAC: MacConkey agar.

**Table 2:** Target genes and primer sequences used in this study.

Targeted Gene	Primer Sequence 5′ to 3′	Amplicon Size	Reference
*Campylobater* spp. (*cadF*)	cadF-F: TTG AAG GTA ATT TAG ATA TG cadF-R: CTA ATA CCT AAA GTT GAA AC	400 bp	[27]
*C. jejuni* (*hipO*)	hipO-F: GAA GAG GGT TTG GGT GGT G hipO-R: AGC TAG CTT CGC ATA ATA ACT TG	735 bp	[27]
*E. coli* (*uidA*)	uidA-F: TAT GGA ATT TCG CCG ATT TT uidA-R: TGT TTG CCT CCC TGC TGC GG	166 bp	[27]
*Listeria* (16S rRNA)	U1: CTC CAT AAA GGT GAC CCT LI1: CAG CMG CCG CGG TAA TWC	938 bp	[28]
*L. monocytogenes* (*hlyA*)	LM1: CCT AAG ACG CCA ATC GAA LM2: AAG CGC TTG CAA CTG CTC	702 bp	[28]
*Salmonella* (random fragment)	ST11: GCC AAC CAT TGC TAA ATT GGC GCA ST15: GGT AGA AAT TCC CAG CGG GTA CTG G	429 bp	[28]
*S.* Typhimurium (*fliC*)	Fli15: CGG TGT TGC CCA GGT TGG TAA T Typ04: ACT GGT AAA GAT GGC T	620 bp	[28]
*Shigella* spp. (*set1A*)	Shig1: TGG AAA AAC TCA GTG CCT CT Shig2: CCA GTC CGT AAA TTC ATT CT	309 bp	[29]
*S. flexneri* (*ipaH*)	ShET1A-F: TCA CGC TAC CAT CAA AGA ShET1A-R: TAT CCC CCT TTG GTG GTA	423 bp	[29]

Footnotes: *cadF*: *Campylobacter* adhesin to fibronectin; *hlyA*: hemolysin A; *set1A*: *Shigella* enterotoxin 1A; *ipaH*: invasion plasmid antigen H; *hipO*: hippuricase; *fliC*: flagellin; *uidA*: beta-glucuronidase.

**Table 3:** Sequencing data output for each sample.

Location	Sample	Total Number of Sequenced Reads
Kampar	Cabbage	34,642,353
	Spinach	37,129,548
	Lettuce	39,626,617
Gopeng	Cabbage	47,778,289
	Spinach	46,992,789
	Lettuce	41,050,773
Ipoh	Cabbage	36,873,538
	Spinach	36,372,744
	Lettuce	32,172,185
Kampar	Raw Chicken Meat	31,217,785
	Cooked Chicken Meat	31,364,518
	Deli Meat	25,829,333
Gopeng	Raw Chicken Meat	32,086,390
	Cooked Chicken Meat	38,798,640
	Deli Meat	50,473,078
Ipoh	Raw Chicken Meat	36,587,717
	Cooked Chicken Meat	64,105,433
	Deli Meat	29,364,161
Kampar	Honeydew	35,030,608
	Papaya	38,853,325
	Watermelon	41,443,821
Gopeng	Honeydew	41,913,941
	Papaya	33,164,209
	Watermelon	29,072,423
Ipoh	Honeydew	31,864,779
	Papaya	30,559,471
	Watermelon	25,019,535

**Table 4:** Detection of mock community in vegetable, meat, and fruit samples using metagenomics analysis and culture-dependent methods.

Detection Method/Sample	Foodborne Pathogen				
	*Escherichia*	*Campylobacter*	*Listeria*	*Salmonella*	*Shigella*
Metagenomics					
Vegetable	+	+	/	+	+
Meat	+	+	/	+	+
Fruit	+	+	+	+	+
Culture and PCR					
Vegetable	+	+	+	+	+
Meat	+	+	+	+	+
Fruit	+	+	+	+	+

Footnotes: +: Detected; /: Not Detected.

**Table 5:** The number of samples detected using a metagenomics analysis and culture-dependent methods.

	Detected with Culture	Not Detected with Culture
Detected with Metagenomics	34 [True Positive (TP)]	1 [False Positive (FP)]
Not Detected with Metagenomics	16 [False Negative (FN)]	84 [True Negative (TN)]

Footnotes: sensitivity = TP/TP+FN; specificity = TN/TN+FP; positive predictive value = TP/TP+FP; negative predictive value = TN/TN+FN.

**Table 6:** Detection of ARGs of *E. coli* ATCC BAA-197 in vegetable, meat, and fruit samples using metagenomics analysis.

Sample	ARGs of *E. coli* ATCC BAA-197						
	*TEM-12*	*sul1*	*AAC(3)-IIe*	*SCO-1*	*APH(3′)-Ia*	*aadA1*	*catA*
Vegetable	/	+	+	+	+	/	/
Meat	/	+	+	+	+	+	/
Fruit	/	+	+	+	+	+	/

Footnotes: *TEM-12:* Extended-spectrum class A beta-lactamase; *sul1:* Sulfonamide-resistant dihydropteroate synthase; *AAC(3)-IIe*: Aminoglycoside N-acetyltransferase; *SCO-1*: Class A beta-lactamase; *APH(3′)-Ia*: Aminoglycoside O-phosphotransferase; *aadA1*: Aminoglycoside nucleotidyltransferase; *catA*: Type A-1 chloramphenicol O-acetyltransferase. +: Detected; /: Not detected.

**Table 7:** Detection of ARGs of *S*. Typhimurium ATCC 700408 in vegetable, meat, and fruit samples using metagenomics analysis.

Sample	ARGs of *S.* Typhimurium ATCC 700408			
	*sul1*	*aadA16*	*floR*	*CARB-2*
Vegetable	+	+	+	/
Meat	+	+	+	/
Fruit	+	/	+	/

Footnotes: *sul1*: Sulfonamide-resistant dihydropteroate synthase; *aadA16*: Aminoglycoside nucleotidyltransferase; *floR*: Chloramphenicol/florfenicol efflux MFS transporter; *CARB-2*: Carbenicillin-hydrolyzing class A beta-lactamase. +: Detected; /: Not detected.

## Data Availability

The metagenomic sequencing results have been submitted to the SRA database under the accession number PRJNA1204389, accessible via the following link: https://www.ncbi.nlm.nih.gov/sra/PRJNA1204389 (accessed on 2 January 2025). The original contributions presented in this study are included in this article and its Supplementary Materials. For further inquiries, please contact the corresponding author.

## Supplementary Materials

The following supporting information can be downloaded at: https://www.mdpi.com/article/10.3390/foods14030352/s1, Figure S1. Rarefaction analysis of the samples (a) Six samples comprising of HM2G, P3I, RC3I, W3I, W2G, H3I and three mock samples O1L, O1CB, O1H (b) Eleven samples comprising of HM3I, CB3I, RC2G, CB2G, W1K, S3I, P2G, P1K, L3I, H2G, C3I. (c) Eight samples comprising of S2G, S1K, RC1K, L2G, HM1K, H1K, CB1K, C2G. (d) Two samples comprising of L1K and C1K. Plotting activity was highest between 0 to 10 million reads (average) before smoothening and completely plateauing. Table S1. Five most abundant bacterial species in vegetable samples. Table S2. Five most abundant bacterial species in meat samples. Table S3. Five most abundant bacterial species in fruit samples.
