# Supplementary material for: Integrating Metagenomic and Culture-Based Techniques to Detect Foodborne Pathogens and Antimicrobial Resistance Genes in Malaysian Produce
Source: Foods. 2025 Jan 22;14(3):352. doi: 10.3390/foods14030352 (PMC11817458; doi:10.3390/foods14030352)
Supplement: Supplementary file 1 [file foods-14-00352-s001.zip › foods-3378962-supplementary.pdf]

Supplementary Materials

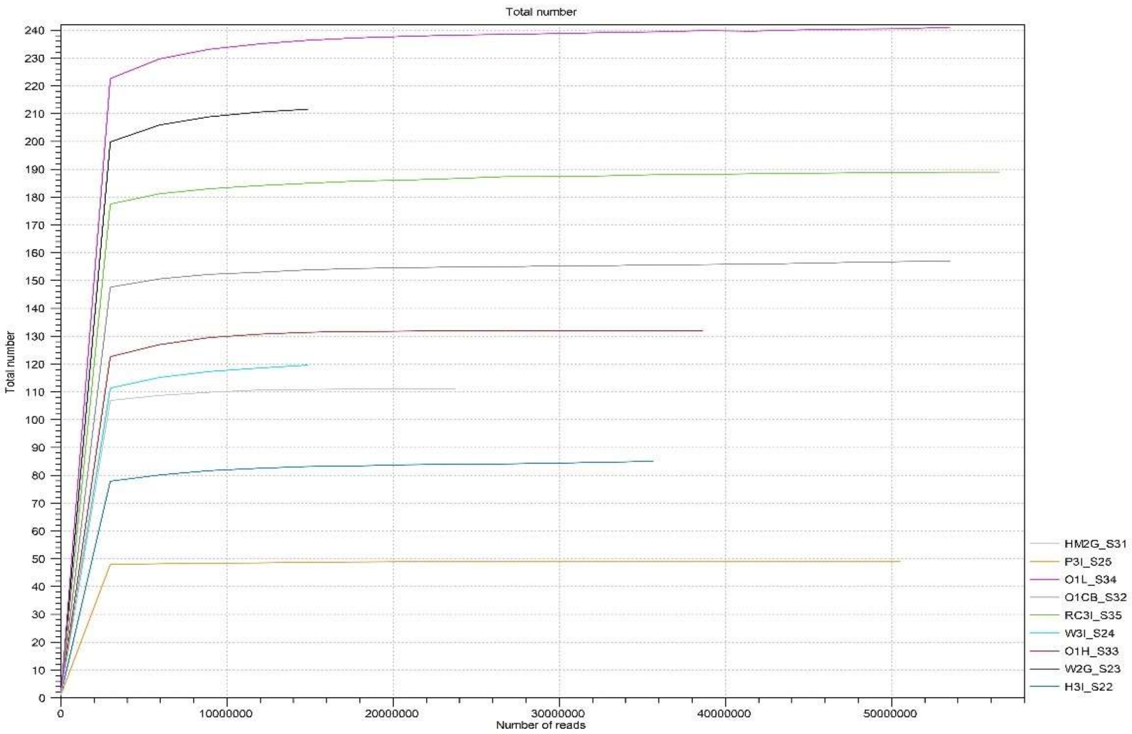

(a)

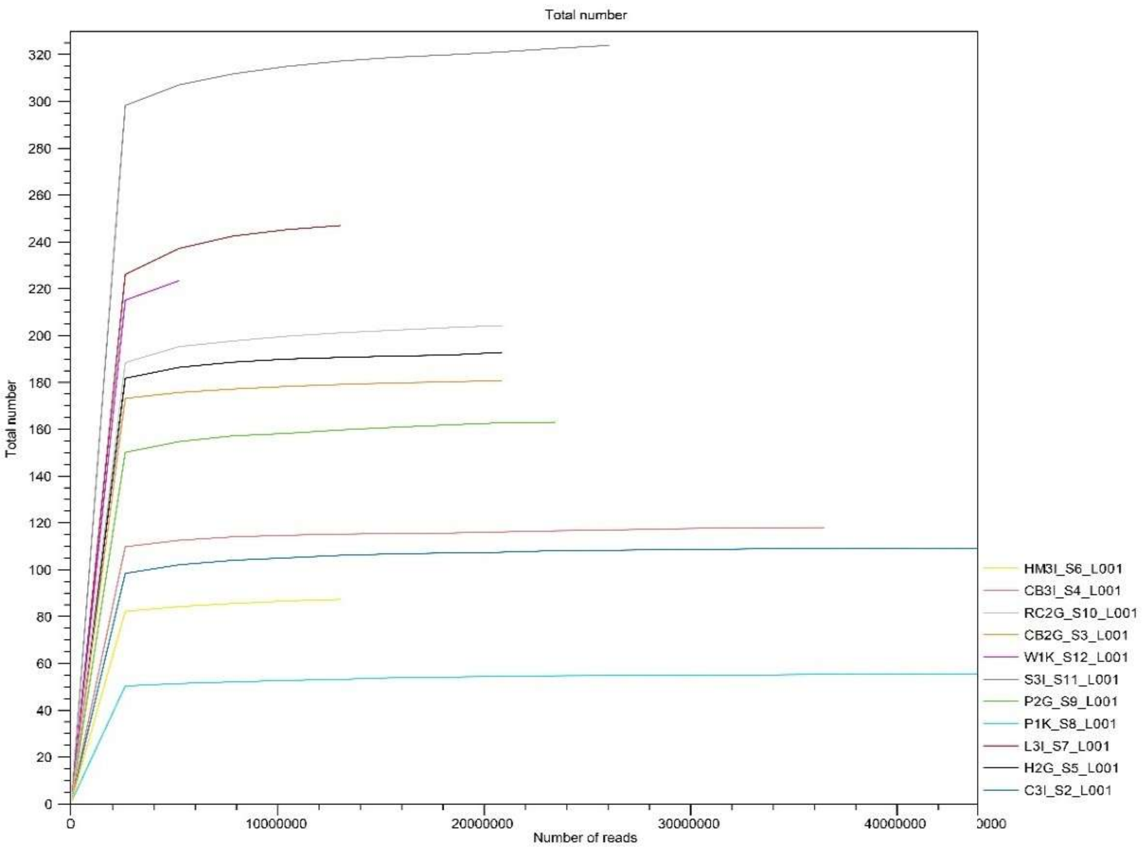
Total number
Total number
Number of reads
0
20
40
60
80
100
120
140
160
180
200
220
240
260
280
300
320
0
10000000
20000000
30000000
40000000
HM3I_S6_L001
CB3I_S4_L001
RC2G_S10_L001
CB2G_S3_L001
W1K_S12_L001
S3I_S11_L001
P2G_S9_L001
P1K_S8_L001
L3I_S7_L001
H2G_S5_L001
C3I_S2_L001

(b)

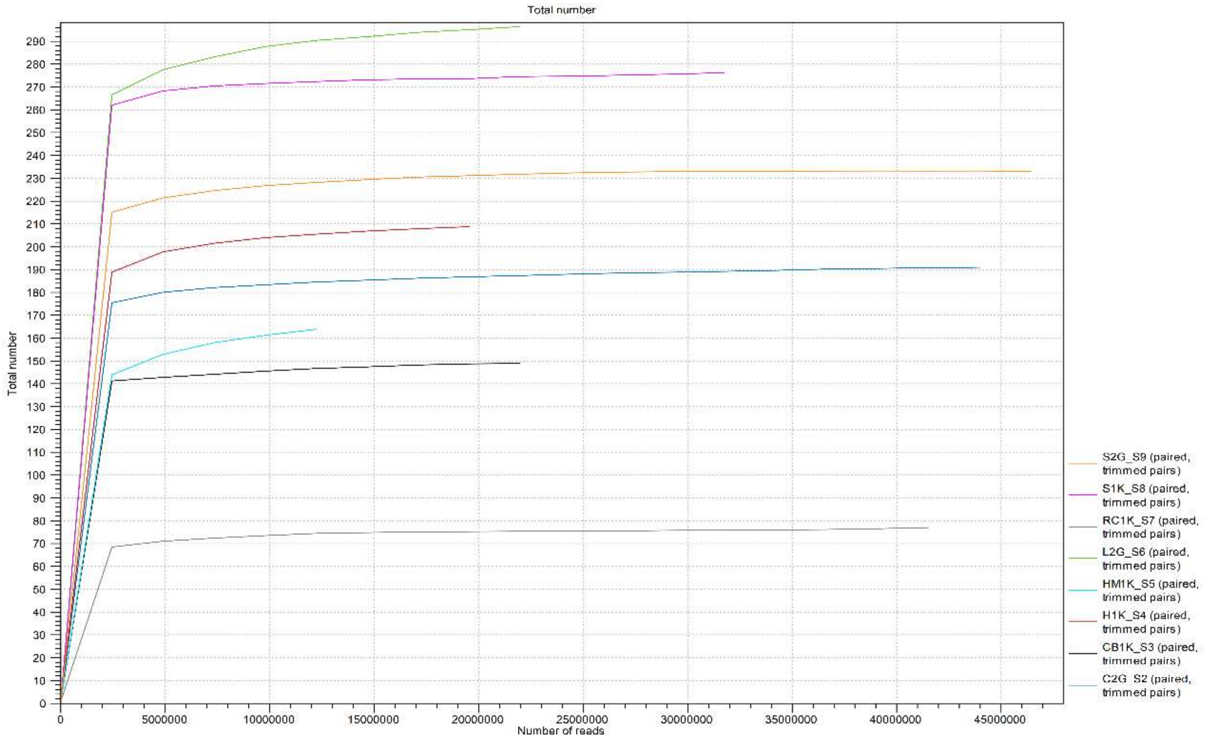
Total number
Total number
Number of reads
S2G_S9 (paired, trimmed pairs)
S1K_S8 (paired, trimmed pairs)
RC1K_S7 (paired, trimmed pairs)
L2G_S6 (paired, trimmed pairs)
HM1K_S5 (paired, trimmed pairs)
H1K_S4 (paired, trimmed pairs)
CB1K_S3 (paired, trimmed pairs)
C2G_S2 (paired, trimmed pairs)

(c)

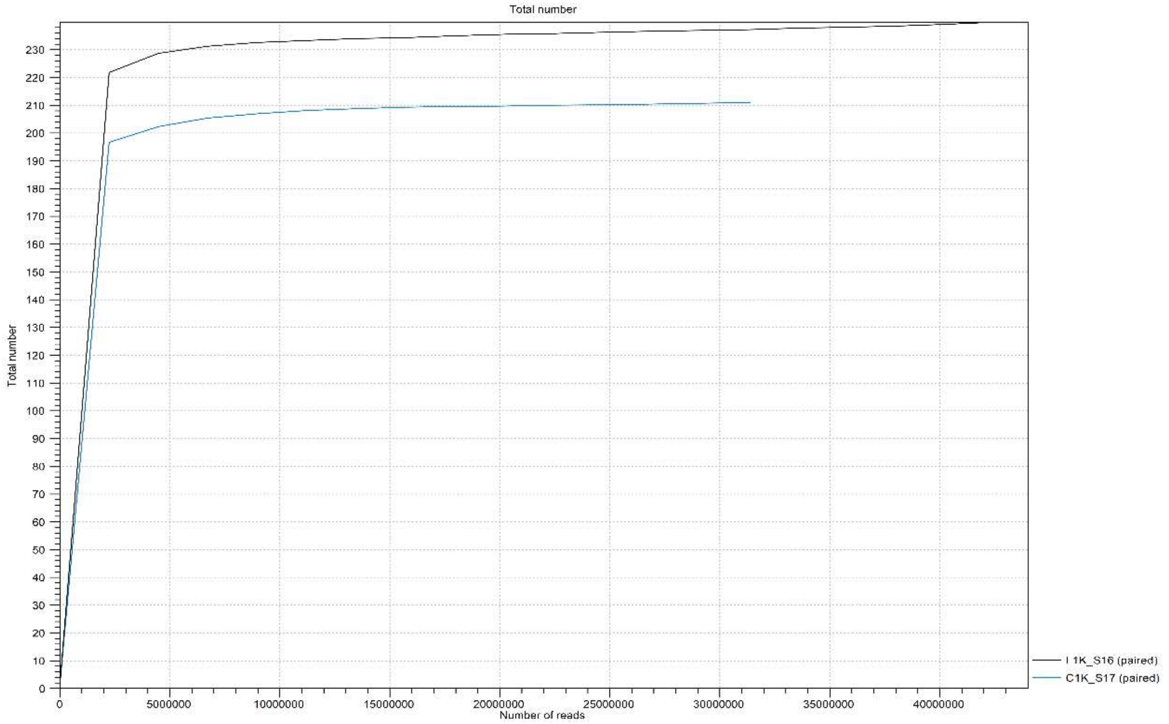

(d)

Figure S1. Rarefaction analysis of the samples (a) Six samples comprising of HM2G, P3I, RC3I, W3I, W2G, H3I and three mock samples O1L, O1CB, O1H (b) Eleven samples comprising of HM3I, CB3I, RC2G, CB2G, W1K, S3I, P2G, P1K, L3I, H2G, C3I. (c) Eight samples comprising of S2G, S1K, RC1K, L2G, HM1K, H1K, CB1K, C2G. (d) Two samples comprising of L1K and C1K. Plotting activity was highest between 0 to 10 million reads (average) before smoothening and completely plateauing.

Table S1. Five most abundant bacterial species in vegetable samples.

Sample	The 5 Most Abundant Bacterial Species				
	1	2	3	4	5
C1K	*Pectobacterium brasiliense*	*Kluyvera ascorbata*	*Acinetobacter pittii*	*Citrobacter freundii*	*Citrobacter portucalensis*
L1K	*Pseudomonas otitidis*	*Pantoea dispersa*	*Lactococcus garvieae*	*Serratia marcescens*	*Klebsiella pneumoniae*
S1K	*Pseudomonas mendocina*	*Pectobacterium brasiliense*	*Arcobacter butzleri*	*Klebsiella aerogenes*	*Comamonas aquatica*
C2G	*Pseudomonas azotoformans*	*Pseudomonas fluorescens*	*Pseudomonas simiae*	*Pseudomonas lurida*	*Enterobacter cloacae*
L2G	*Leclercia adecarboxylata*	*Pseudomonas fulva*	*Pectobacterium brasiliense*	*Pectobacterium polaris*	*Pseudomonas oryzihabitans*
S2G	*Aeromonas hydrophila*	*Enterobacter cloacae*	*Aeromonas caviae*	*Enterobacter roggenkampii*	*Pseudomonas putida*
C3I	*Pantoea agglomerans*	*Pantoea ananatis*	*Pectobacterium carotovorum*	*Pseudomonas lurida*	*Pectobacterium brasiliense*
L3I	*Pantoea rwandensis*	*Acinetobacter baumannii*	*Enterobacter cloacae*	*Pectobacterium brasiliense*	*Pseudomonas putida*
S3I	*Pectobacterium brasiliense*	*Leclercia adecarboxylata*	*Aeromonas caviae*	*Pectobacterium carotovorum*	*Enterobacter cloacae*

Table S2. Five most abundant bacterial species in meat samples.

Sample	The 5 Most Abundant Bacterial Species				
	1	2	3	4	5
CB1K	*Aeromonas veronii*	*Aeromonas salmonicida*	*Shewanella baltica*	*Buttiauxella agrestis*	*Aeromonas hydrophila*
HM1K	*Kurthia zopfii*	*Myroides phaeus*	*Brochothrix thermosphacta*	*Providencia rettgeri*	*Providencia stuartii*
RC1K	*Vibrio fluvialis*	*Klebsiella pneumoniae*	*Shewanella algae*	*Acinetobacter baumannii*	*Proteus mirabilis*
CB2G	*Aeromonas veronii*	*Citrobacter freundii*	*Klebsiella aerogenes*	*Shewanella baltica*	*Aeromonas caviae*
HM2G	*Lactococcus lactis*	*Leuconostoc citreum*	*Leuconostoc carnosum*	*Enterococcus gilvus*	*Leuconostoc mesenteroides*
RC2G	*Klebsiella pneumoniae*	*Lactococcus lactis*	*Pantoea dispersa*	*Weissella ceti*	*Acinetobacter calcoaceticus*
CB3I	*Shewanella baltica*	*Aeromonas veronii*	*Aeromonas salmonicida*	*Pseudomonas fragi*	*Aeromonas hydrophila*
HM3I	*Providencia rettgeri*	*Leuconostoc mesenteroides*	*Providencia rustigianii*	*Providencia stuartii*	*Leuconostoc carnosum*
RC3I	*Klebsiella pneumoniae*	*Acinetobacter johnsonii*	*Pseudomonas putida*	*Pseudomonas monteilii*	*Acinetobacter tandoii*

Table S3. Five most abundant bacterial species in fruit samples.

Sample	The 5 Most Abundant Bacterial Species				
	1	2	3	4	5
W1K	*Weissella ceti*	*Tatumella ptyseos*	*Enterobacter hormaechei*	*Leuconostoc lactis*	*Leuconostoc garlicum*
H1K	*Klebsiella variicola*	*Pantoea agglomerans*	*Lactococcus lactis*	*Leuconostoc lactis*	*Kosakonia cowanii*
P1K	*Enterobacter hormaechei*	*Pantoea dispersa*	*Leuconostoc lactis*	*Kosakonia cowanii*	*Leuconostoc garlicum*
W2G	*Leuconostoc lactis*	*Enterobacter cloacae*	*Acinetobacter seifertii*	*Enterobacter roggenkampii*	*Klebsiella quasipneumoniae*
H2G	*Enterobacter asburiae*	*Acinetobacter seifertii*	*Acinetobacter baumannii*	*Enterobacter roggenkampii*	*Enterobacter cloacae*
P2G	*Enterobacter cloacae*	*Leuconostoc lactis*	*Enterobacter hormaechei*	*Acinetobacter baumannii*	*Acinetobacter seifertii*
W3I	*Enterobacter cloacae*	*Leclercia adecarboxylata*	*Pseudomonas putida*	*Enterobacter hormaechei*	*Pseudomonas monteilii*
H3I	*Klebsiella michiganensis*	*Enterobacter roggenkampii*	*Klebsiella variicola*	*Klebsiella oxytoca*	*Enterobacter cloacae*
P3I	*Rouxiella badensis*	*Enterobacter hormaechei*	*Lactococcus lactis*	*Leuconostoc lactis*	*Klebsiella pneumoniae*
